# Implications of the Ebola virus disease outbreak in Guinea: Qualitative findings to inform future health and nutrition-related responses

**DOI:** 10.1371/journal.pone.0202468

**Published:** 2018-08-23

**Authors:** Stephen R. Kodish, Fabian Rohner, Jean-Max Beauliere, Mamady Daffe, Mohamed Ag Ayoya, James P. Wirth, Ismael Ngnie-Teta

**Affiliations:** 1 GroundWork, Fläsch, Switzerland; 2 United Nations Children’s Fund (UNICEF), Conakry, Guinea; 3 Ministry of Health, Conakry, Guinea; University of Virginia, UNITED STATES

## Abstract

**Introduction:**

Due to the close relationship between EVD and nutrition, the humanitarian community implemented various nutrition-specific and -sensitive interventions to stem the Ebola Virus Disease (EVD) outbreak in West Africa. Little, however, is known about stakeholder and community members’ perspectives toward this response in Guinea. Therefore, we aimed to firstly understand how EVD may have influenced the nutrition situation; and secondly to assess the perceived acceptability and effectiveness of the nutrition response.

**Materials and methods:**

Using 27 in-depth interviews conducted in April–May 2016, this descriptive, qualitative study had three iterative phases in an emergent design. *Phase 1* explored the perceptions of 11 high-level policy and management staff. *Phase 2* assessed the views of 16 community members, survivors, and front-line workers. *Phase 3* compared the qualitative findings to relevant nutrition indicators from secondary data for final interpretations. A systematic, team-based coding approach using Dedoose software identified key themes during textual analysis.

**Results:**

Overall, several plausible pathways through an interrelated network of bio-social factors help describe EVD impacts on the nutrition situation of Guinea. At a basic level, complex social dimensions of health, response unpreparedness, and market disruptions were perceived to be major determinants affecting the nutrition situation, especially among IYC. At an underlying level, household food security was negatively impacted, along with weakened care-seeking practices, IYC feeding practices, and coping strategies. Consequently, treatment coverage for childhood illnesses and IYC diets were negatively impacted during the outbreak. In hindsight, most participants had positive perceptions toward the overall EVD response, but described salient considerations for improving upon this nutrition response during future outbreaks.

**Discussion:**

This study highlighted the complex web of inter-related factors through which EVD was perceived to impact the nutrition situation in Guinea. Considering the multi-level social and behavioral dimensions of health and nutrition is critical for effectively responding to infectious disease outbreaks.

## Introduction

In 2013, the already resource-constrained health systems of West Africa were confronted with an infectious disease outbreak that required a global response. The first case of Ebola Virus Disease (EVD) was observed in Guinea in December 2013 and officially declared by March, 2014 [1]. Rapidly, the outbreak spread throughout Guinea, Sierra Leone and Liberia; individual cases were reported for several other countries as well, but could be contained. Both community members and health workers, including front-line treatment and care staff, shouldered the substantial proportion of the burden, being asked to treat an epidemic where risks for contagion as well as fatality were high [2, 3]. The burden of disease from EVD was heavy, with more than 3,800 cases and 2,500 deaths as a direct consequence of the virus in just two years in Guinea alone [4]. Since then, there have been important lessons learned from both the outbreak and the response to it across myriad health sectors, nutrition notwithstanding.

Ebola overtly impacts a person’s nutritional status through severe dehydration and nutrient loss from bleeding, vomiting and watery diarrhea, as well as gastrointestinal pain contributing to reduced appetite, nausea, and anorexia [5, 6]. Even during recovery, EVD survivors have trouble returning to pre-disease health and nutritional status due to overall lethargy, joint pains, decreased appetite, as well as psychosocial conditions on physical health [7, 8]. Indirectly, nutrition-related consequences of the infection are just as concerning for community health. For instance, children under five years, an already vulnerable population, become more susceptible to acute malnutrition when caregivers alter infant and young child feeding (IYCF) practices to cope with transmission risk through breastmilk [9]. Disruptions to services at health centers increase this susceptibility as therapeutic nutrition care becomes more difficult to access as it is commonly provided in those centers. Also, community quarantines may prevent the provision of key nutrition interventions in affected communities, such as community-wide vitamin A supplementation. Appropriate nutrition actions are therefore one of the primary keystones in appropriate EVD treatment, care, and recovery, not only for individual patients but also communities.

In response to the EVD outbreak in Guinea, donors in support to Government, increased funding for enhanced logistics and medical supplies (e.g. protective gears, ambulances, motorbikes etc…), and provided targeted technical capacity [10]. Empirically, evidence suggests that the response in Guinea eventually was successful in stemming the outbreak, although critiques related to a limited capacity in fragile health systems, slow response times, insensitivities toward community member views and culture, and lack of coordination are just some that have emerged in hindsight [11–13]. Specific sectors, such as nutrition, provided nutrition-specific support during patient care, treatment, and recovery, including but not limited to food commodities for food insecure households, specialized nutritious foods to prevent and treat acute malnutrition cases, and ready-to-use infant formula for infants who needed replacement feeding [14–16]. Preventatively, increased levels of screening for acute malnutrition were accompanied by communications aimed at enhancing nutrition-related practices in the context of the outbreak [15].

Nutrition-related social and behavior change communications (SBCC) were also delivered through community sensitization activities, mass media, and interpersonal messages delivered through community health workers and front-line care staff. Interim guidelines were developed by the global community to aid in-patient and community health workers in passing appropriate health and nutrition messages in the context of EVD. Specifically, interim guidelines for *Nutritional Care of Children and Adults with Ebola Virus Disease in Treatment Centers* [17], *Infant Feeding in the Context of Ebola* [16], *and Clinical Care for Survivors of Ebola Virus Disease* [18], were created specifically for use during this outbreak. Despite these many nutrition actions, it is unclear to what extent these response options were accepted and utilized usefully in Guinea.

Reports have outlined specific nutrition-related outcomes (e.g., number of EVD patients having received nutrition support during treatment), but little work has been conducted to explore acceptability and perceived effectiveness of the inputs, including the interim guidelines. So despite its primary importance in both EVD care and recovery, there is very limited knowledge about perceptions of the quality of nutritional support within the larger Ebola response. And without a clear understanding of the most effective nutrition-related response options available during infectious disease outbreaks, future responses in Guinea and elsewhere may be hampered by a paucity of effective strategies and tools at their disposal for timely response.

Therefore, this study first sought to understand how the Ebola outbreak may have impacted infant and young child nutrition in Guinea. Second, we aimed to understand how stakeholders at multiple different levels perceived the acceptability and effectiveness of the nutrition-specific response during this outbreak to draw lessons learned and make recommendations for consideration in future similar scenarios.

## Materials and methods

### Research design

This qualitative study had three-phases and was iterative in nature. Findings from each phase built off those of the previous phase in an exploratory and inductive methodological approach [19–21]. **Phases 1** and **2** explored the perceptions of multiple, different stakeholder groups to describe the impacts of the Ebola outbreak on health and nutrition programming from their perspectives. **Phase 3** was used to examine secondary data, comparing the nutrition impacts during the time of EVD outbreak to those found in qualitative data of phases 1 and 2.

### Sampling

#### Data collection

Primary data collection occurred during April and May 2016, over two iterative phases, by Guinean research assistants trained in qualitative interviewing skills. All participants for semi-structured interviews were purposefully selected through a criterion-based sampling strategy [22]. This type of strategy is used in qualitative research as a way to deliberately select particular settings, persons, or activities in order to provide information that cannot be obtained readily otherwise [21]. Data were collected until data saturation, or repetition of information, was reached among key themes [23]. Phases of data collection are described below.

#### Phase 1. Understanding policy and management perceptions

In-depth interviews were conducted first among ‘key informants’ using a semi-structured interview guide “S1 File” covering 9 domains (Table 1). We interviewed in-country stakeholders from the primary organizations that were involved in addressing nutrition as part of the Ebola response, including but not limited to Government officials, United Nations bodies, non-Government organizations (NGOs), and Medical Facilities Administration. We collected data from both decision makers and managers.

**Table 1:** Semi-structured interview guide content.

	Key Informant Guide Content	Informant Guide Content
**1.**	Ebola impact (general impact)	Ebola impact (general impact)
**2.**	Ebola impact (on organization)	Perceptions of nutrition care to Ebola patients
**3.**	Ebola impact (on health system)	Perceptions of nutrition support to Ebola survivors
**4.**	Ebola impact (on nutrition services within stakeholder organization)	Ebola impact on infant and young child feeding
**5.**	Quality of support for nutritional health	Recommendations and lessons learned
**6.**	Nutritional support using interim guidelines	
**7.**	Coordination and information sharing	
**8.**	Recommendations and lessons learned	

#### Phase 2. Exploring community and front-line health worker perceptions

Community members were then interviewed as ‘informants’ using a second semi-structured guide “S2 File” with 6 domains.These individuals included community leaders, EVD survivors, family members of EVD victims, midwives, and health workers who were at the frontlines of the response.

All interviews were digitally recorded in French or a local language, each lasting approximately between 30–60 minutes, in convenient, private locations chosen by participants. Field notes were taken immediately following interviews by interviewers for reflection on key questions and commentary on transcript quality or sampling considerations [19].

#### Phase 3. Synthesizing findings vis-à-vis relevant indicators of secondary data

Secondary data sources with nutrition-related data from the Ebola response were collected and reviewed for relevant information to triangulate qualitative data collected in phases 1 and 2. Secondary data originated from sources provided by UNICEF in the form of unpublished reports, as well as a web-based literature search using keywords around Ebola and nutrition.

### Data analysis

The textual analysis followed a systematic and multi-step process. After interviews were completed, digital recordings were transcribed in the actual language of the interview and subsequently translated into English. Spot checks on approximately 15% of the interviews were completed by one bilingual (French and English) study team investigator to ensure accuracy of translations and completeness of transcripts when compared to their digital recordings.

The textual data were then uploaded into Dedoose software [24] for data management and analysis. Concurrently, research team members developed an initial codebook containing 43 codes within 9 overarching descriptive domain categories. The codebook contents were developed based on the interview guide content, study objectives, and research questions. As part of the codebook development process, user-friendly definitions and detailed instructions were included in the analytic framework in order to ensure its appropriate use among multiple different users/coders [22, 25].

To establish inter-coder reliability among the pair of primary coders, one person initially coded approximately 25% (7 transcripts) of the data set. Then the other member blindly re-coded those same transcripts in order to estimate rater agreement across many codes using Kappa scores ranging from 0 (*agreement no better than chance*)– 1 (*perfect agreement*) [26]. This first effort yielded a score of 0.54 suggesting only “fair” reliability based on established cutoffs [27]. Before proceeding, adjustments were made to the codebook guidelines and coding efforts were reviewed as a larger team to improve more consistent application of codes [28]. Second and third reliability tests yielded scores of 0.81 and 0.94, respectively, and were both considered “excellent” [27]. Only then was the remainder of the data set coded by both team members using a finalized codebook with 42 codes and 7 descriptive categories. Coding was completed following an inductive approach drawn from procedures of Grounded Theory [29]. In total, the 42 codes were applied 643 times to the overall data set.

After coding, we extracted key themes by participant group (i.e., key informants vs. informants). Then, comparisons within and between groups were made. Salient themes were identified based on both the frequency of mention and importance of content for addressing the study objectives [30]. Qualitative findings were then interpreted vis-à-vis the secondary quantitative data extracted from grey literature for final interpretation and presentation of results.

### Ethical approval

Qualitative research approval was given by the Guinea Ministry of Health, which has the authority to approve human subjects research of minimal to no risk. Prior to interviewing, all participants provided oral informed consent prior and no identifiers were collected as part of study procedures.

## Results

### Summary of socio-demographics

Overall, 11 key informants and 16 informants (*n* = 27) participated in this study. Most participants were community members or front-line care staff (59.3%), whereas other were high- and mid-level stakeholders (40.7%) (Table 2). Overall, two thirds of our sample represented community-level voices and perspectives, of which 44% female and 56% male.

**Table 2:** Socio-demographic characteristics of study sample.

**All participant characteristics**		
	No. of participants, *n*	27
	Female, *n* (%)	12 (44.4%)
**Key Informant characteristics** (policy and managerial staff members)		
No. of key informants, *n* (%)		11 (40.7%)
Type of organization represented, *n* (%)		
	Government/Policy	4 (36.4%)
	United Nations	4 (36.4%)
	Hospital Management	2 (18.2%)
	Non-government organization (NGO) management	1 (9.1%)
Years spent in professional role, median(min, max)		3 (1–10)
**Informant characteristics** (community members and front-line workers)		
No of informants, *n* (%)		16 (59.3%)
Type of informant, *n* (%)		
	Health workers (medical doctor, nurse, front-line staff, midwife)	7 (43.8%)
	Household or community members of Ebola victim	6 (37.5%
	Community leaders (village headmen)	2 (12.5%)
	Survivors of EVD	1 (6.3%)
**Geographic regions represented, *n* (%)**		
	Conakry	14 (51.9%)
	Kindia	6 (22.2%)
	Nzérékoré	5 (18.5%)
	Labé	1 (3.7%)
	Faranah	1 (3.7%)

Organizationally, the 11 key informants represented perspectives from a range of bodies, including those of the Government/Policy, United Nations, Hospital Management, and NGOs. The 16 informants provided perspectives from 7 front-line health workers, 6 household or community members of Ebola victims, 2 community leaders, and 1 Ebola survivor. Geographically, individuals representing both community and professional roles participated from across 5 of Guinea’s 8 administrative regions that were most impacted by Ebola.

### Perceptions of overall Ebola impact

**Fig 1:**
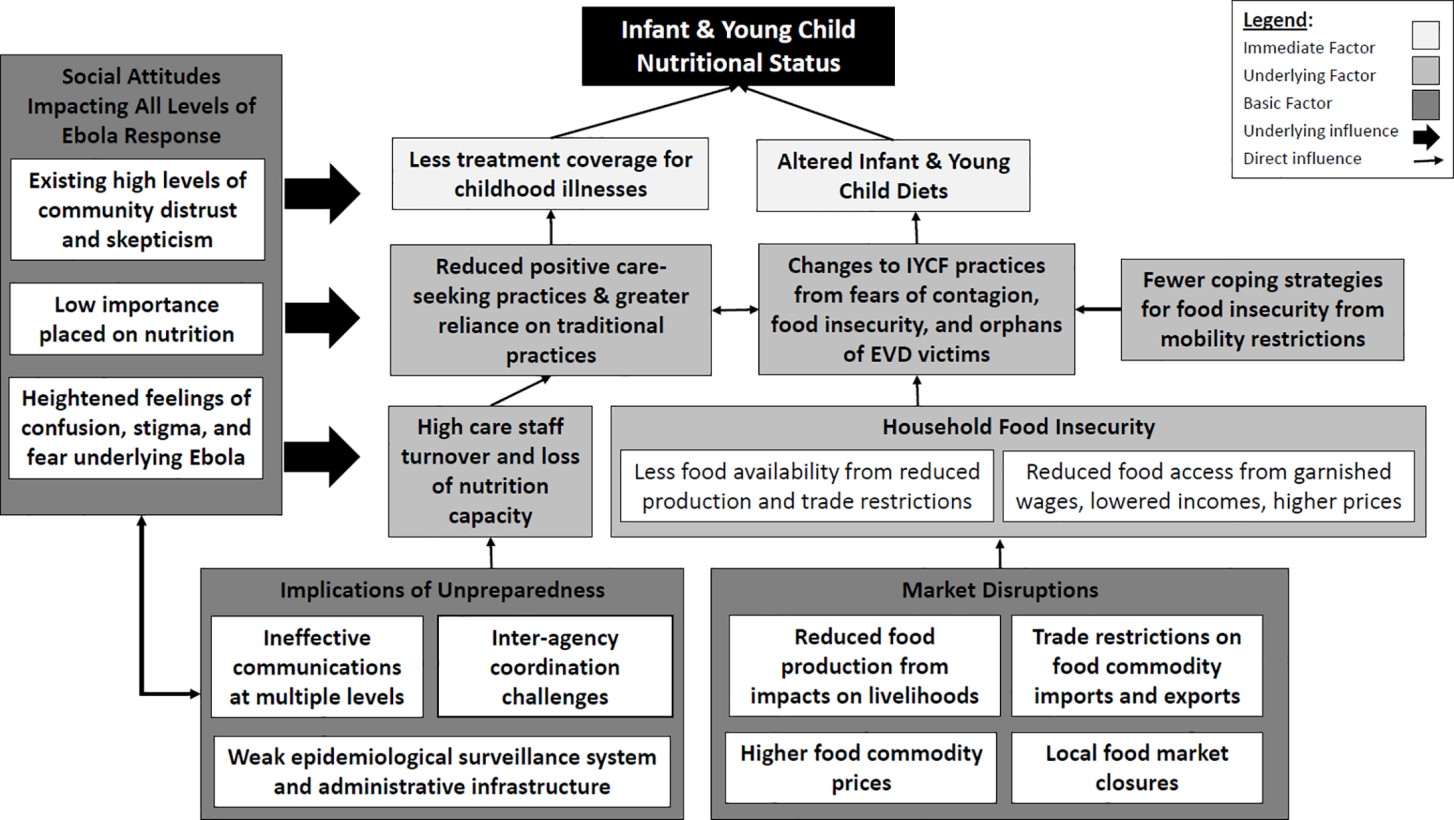
Plausible pathways explaining Ebola indirect impacts on infant & young child nutrition.

Overall, participants had positive perceptions toward the overall EVD response. However, there were three primary themes that emerged as specific areas of improvement and can be represented as an overall figure depicting qualitative findings (Fig 1): unpreparedness, market disruptions, and social attitudes.

#### Implications of unpreparedness

First, key informants highlighted the initial ***inter-agency coordination challenges***, which reportedly slowed down the overall response and stemmed from having “*no response plan*.” Second, key informants acknowledged Guinea’s resource-constrained health system with ***weak epidemiological surveillance system and administration infrastructure*** to respond to this type of disease outbreak. Key informants emphasized the importance of the international community in supporting Government through resource mobilization indicating that response quality was largely “*an issue of means*”.

Third, while key informants praised the importance of improved communications over time, they also explained that initially, there were ***ineffective communications at multiple levels***. At the national level, key informants discussed the delicate nature of declaring a national outbreak, citing potential consequences resulting from decreased foreign investment and potential disruption of social calm, affecting the overall well-being of the state. At the organizational level, data indicate there was weak coordination leadership among actors that typically work within the same health system but not often in tight collaboration with one another. And at the community level, initial messages had unintended consequences. For instance, some communications intended to promote care-seeking behavior had the opposite effect based on the framing of the messaging. Community members were reportedly unclear why they were told to go to the hospital when Ebola was thought to be incurable.

*“So, at the start, it was not easy, even at the communication level*. *From the moment we started saying that it (Ebola) was not a disease we could cure, it created a psychotic atmosphere among the population. So then, people said, ‘Since it’s a disease that cannot be cured, why do you want us to go to the hospital?’”*

                                        -Key informant, Nutrition Staff, Conakry

Similarly, there was confusion around the response slogan, “*End Ebola in 60 Days*,” which community members reportedly perceived to mean that the biomedical community had a cure but just were not providing it to them at that time.

#### Social attitudes impacting all levels of Ebola response

Thus, ***ineffective communications at multiple levels*** reportedly contributed to ***heightened feelings of confusion*, *stigma*, *and fears underlying Ebola***. They also exacerbated ***existing levels of distrust and skepticism*** among the local community toward the biomedical community, contributing to ***reduced positive care-seeking practices and greater reliance on traditional practices***.

While these challenges were not explained by community members in the context of nutrition specifically, recurring themes indicated that the Ebola challenges were in fact linked to nutrition at multiple levels of influence:

“*Everyone was disturbed, at work…everyone no longer had that courage, everyone was paralyzed…the farmer could not properly do his farming, the fisherman, as well, could not go to fish…even government workers (couldn’t work). Because the epidemic was here, there was no more trust between the people, for example…when someone felt sick it was difficult for him to go to the hospital…they felt like if they go to the hospital and it happens to be this disease (Ebola), then it’s over for him…even between husbands and wives there was this suspicion. When the epidemic began to disappear, people started their old behaviors, started to go and work, some started farming, but the impact was there because people could not afford things. The whole year of 2015, the impact was there… before Ebola, the number of children that had malnutrition… (compared to) after Ebola, (well) the number tripled up…it was severe malnutrition*.”

                                        Informant, Community Member, Nzérékoré

The community member above summarized several effects of Ebola at basic, underlying, and immediate levels of nutrition. Despite this finding, key informants explained that there was a ***low importance placed on nutrition*** during the outbreak among both health professionals and community members.

### Nutrition-specific challenges in the context of Ebola

The nutrition sector faced major challenges and an increased acute malnutrition caseload, according to interviews with front-line workers. National-level quantitative data of actual caseload numbers make it difficult to draw sound conclusions due to intermittent reporting during the 2014–2015 outbreak period [31]. Data from the Labé region showed a continuous increase in Outpatient Therapeutic Programme (OTP) admissions through August 2014, which was in the early stage of the peak of EVD. Then during the peak and following months, a substantial decrease in OTP caseload was observed, as well as a simultaneous drop in both number of nutrition health workers and consultations given [32].

All participants explained that the relatively ***low importance placed on nutrition*** at both systems and community levels, particularly in context of infectious disease, was a likely contributing factor to Ebola impacts on nutrition. During the outbreak, key informants explained that they had “*very few tools for nutrition sensitization*” and a ***large turnover of care staff and loss of nutrition capacity*** due in part to the ***heightened feelings of confusion*, *stigma*, *and fear underlying Ebola***.

“*The impact of the Ebola Virus Disease on our (nutrition) department was in two ways: First, it is the nursing staff that was stigmatized. Some died and others were hospitalized. Others…they stopped coming. Then…in 10 service providers, only 2 were coming. That was a burden. (Second), the few patients that were here were (as a result) a heavy burden for the few staff that was coming*.”

                                        Key Informant, Hospital Management, Conakry

In addition to staff and organizational constraints impacting care practices, study participants explained that household food security was also heavily impacted during the outbreak.

#### Household food insecurity stemming from market disruptions

Less food availability. Ebola disrupted national and local food markets in several ways, according to participants. First, participants explained that Ebola ***reduced food availability from impacts on livelihoods*** because many individuals, particularly women, could no longer engage in typical agricultural practices due to falling ill. Second, the national ***trade restrictions on food commodity imports and exports*** also contributed to less availability of food, explained key informants. The review of secondary data also supports this relationship between trade bans and decreased food availability, particular in the Nzérékoré region where compared to the previous year, 24% and 51% of the respondents from non-affected and affected areas, respectively, had to modify the ways they typically procured basic food items due to such market disruptions [33].

Reduced food access. Key informants explained that ***higher food commodity prices*** increased because of ***market disruptions***.

*“Here (in Conakry) we used to pay 40*,*000 to 50*,*000 GNF* [4.4–5.5 USD]*for a daily meal and we would send some to our friends*, *but finally (because of the outbreak) it was necessary to pay over 100*,*000 GNF* [11.0 USD] *for the same meal*, *because of price increases of everything in the markets*.*”*

                                        Key informant, Hospital Supervisor, Conakry

Empirically, secondary data show that some but not all commodity prices increased nationally. For instance, while rice prices increased, those of corn, yam, and cassava declined. For the most affected areas, substantial price drops occurred from a lack of demand stemming from people’s lack of access, as well as fear and stigma associated with gathering in crowded market areas [33–35].

Qualitative data revealed also how individual livelihoods were disrupted. Garnished wages and lowered incomes, coupled with higher prices, meant reduced food access for households. Ebola-affected areas, where food commodities once were sold, saw entire ***local food market closures*.**

“*It was difficult…this is a zone that produces a lot of pineapples and tomatoes. When people knew that there was Ebola into our zone, there was no business. The women that used to sell by the road side were not selling anymore because vehicles were not stopping anymore due to the Ebola epidemic*.”

                                        Informant, Community Member, Friguiagbe

Coupled with local market disruptions, there were also food access challenges stemming from the mobility restrictions placed upon households where one or several members had been diagnosed with Ebola. Households had ***fewer coping strategies for food insecurity from mobility restrictions***. Typical strategies, such as inter-household food sharing (i.e., food trade between households), were made very difficult with quarantine sanctions imposed.

“*First of all, we went several months without going to markets, our markets were spoiled, and it is through the small villages that we (typically) did transactions so we could get what we needed to eat…but these village members were forbidden from moving from one place to another…we had nothing…there were people who could hardly get anything to eat*.”

                                        Informant, Community Leader, Beyla

#### Changes to IYCF practices

Increased levels of household food insecurity and fewer coping strategies therefore were factors that contributed to *changes to IYCF* practices because many households now lacked the ability to access nutritious complementary foods and were in positions where others could not help due to the mobility restrictions put in place. And at the individual level, infants and young children whose mothers had been afflicted with Ebola were advised to no longer engage in exclusive or continued breastfeeding. Also, informants ascribed underlying ***fears of contagion*** for preventing typical community interactions related to food, explaining that improving food security was one of the primary areas were the nutrition response was so helpful because organizations helped where the community could not.

The ***ineffective communications at multiple levels*** had consequences for not only care-seeking but also IYCF practices. Data suggested that traditional food-related practices (e.g., heavy reliance on energy-dense, non-nutritious staple foods) were particularly difficult to change even in the face of increased household food insecurity and malnutrition during the outbreak, underscoring the ***low importance placed on nutrition*** and reinforcing the importance of the ***underlying distrust and skepticism*** toward the biomedical community during the outbreak.

### Perceptions of the nutrition response

In response to the challenges described above, the humanitarian nutrition and food security response provided food assistance and social and behavior change communications. We found both barriers and facilitating factors related to their acceptability and utility during the outbreak (**Table 3**).

**Table 3:** Salient themes toward nutrition response efforts during Ebola in Guinea.

**Nutrition and food assistance**	
**Initial Barriers**	**Facilitating Factors and Lessons Learned**
Underlying levels of distrust, misinformation, fear, confusion impacted initial acceptance of products–perceptions of Ebola in food	Products were highly acceptable with high demand over time when trust had been built up through improved sensitization efforts
Frequent stock-outs of specialized nutritious foods were reportedly common	Increased funding during outbreak allowed organizations to afford expensive food and nutrition products
Sharing of specialized nutritious foods was suspected at the household level among care workers	Food and nutrition provisions increased community confidence by illustrating strong donor support
	Messaging by care staff to community members reportedly was useful for improving usage of specialized nutritious foods
**Social and behavior change communications**	
**Initial Barriers**	**Facilitating Factors and Lessons Learned**
Underlying levels of community distrust, misinformation, fear, confusion around Ebola outbreak limited acceptability of messaging	Combination of mass media plus interpersonal messages was important for reaching different audience segments
Traditional dietary practices around infant and young child feeding were difficult to change	Taking time for pilot testing is critical for identifying inappropriate messages, even if slower
High illiteracy required special considerations for messaging	Using the traditional health community, including community health agents, midwives, and wet nurses was important for improving IYCF
Weak coordination meant that early communication efforts were hampered with care staff not always aware that guidelines were available	Using familiar, trusted channels for passing interpersonal messages was critical for buy-in and acceptance of new nutrition behaviors
Unintended consequences of developing messaging without prior testing negatively impacted care-seeking practices	Community-level committees that were formed successfully connected biomedical and traditional health communities and are still in place today
Limited individuals with enough social and behavioral expertise and capacity for developing culturally-appropriate and effective messaging	Community-level trainings were effective for training on health and nutrition guidelines in this context

#### Nutrition and food assistance

To address household food security, SuperCereal+ and other commodities, including rice, lentils, peas, rice, beans, oil, salt, and sugar, were provided. Specialized nutritious foods, including ready-to-use therapeutic foods (RUTF) for severe acute malnutrition and replacement milks for children who could no longer breastfeed, were also provided. Initially, community fear and distrust were barriers to utilizing these food provisions, with perceptions that foods given out by health staff contained Ebola. One community member explained, “*…(due to the) loss of lives*, *so I didn’t have the courage to eat (foods given out)*.”

Participants explained that more intensive and appropriate sensitization efforts increased the acceptability of the food assistance over time. The RUTF was highly acceptable by community members and care staff, with reportedly high demand but frequent stock outs. “*Sometimes the quantity that was demanded by the population was often more than the stock*,” said a community member from Friguiagbe.

Community members without children or who had not been directly impacted by EVD were reportedly requesting the RUTF, an indication of its high acceptability. Care staff explained that they were promoting it as a “*food with medicine*” to reducing suspected intra-household sharing at the expense of intended beneficiaries. Key informants noted the high costs of infant formulas for replacement feeding, which was made affordable during the outbreak due to increased resources from donors, a facilitating factor to improved response efforts.

Like most aspects of the response, acceptability around the food assistance improved over time, which was eventually an important contributor to increased community confidence and trust toward the outbreak response at large. Food assistance was perceived to be a very direct and tangible link between donors and community support.

“*The foods that organizations were supplying to the community, especially to victims of Ebola, started building confidence in the community and the people. But at the beginning, it was not easy. It was very difficult. The fact that the donors came closer to the community and to the people materially and financially…sensitization had already started. The community started to believe and trust the donors and their implementing partner …*”

                                        Informant, Community Leader, Kindia

#### Social and behavior change communications (SBCC)

SBCC were implemented to improve both behaviors of care staff and community members during the response.

Interim guidelines for organizations and front-line care staff. As part of the larger multi-level communications strategy, interim guidelines were provided to organizational care staff. Key informants offered mixed opinions toward the usefulness of the guidelines, indicating not being aware or not using them. However, some key informants did indicate that while they were useful in theory, some of the recommended practices contained within them were difficult to implement in practice. For instance, participants explained that separating one’s baby from his/her mother, especially during an outbreak, was too challenging and alternative strategies needed to be provided.

“*In theory, it was easy but in practice, it was not easy. For example, isolating a child from his mother is not an easy thing to do in communities where we know that there is a reinforced bond between the child and his/her mother so it is not easy to apply that guideline*.”

                                        Key Informant, Nutrition Coordinator, Conakry

Further, participants explained that such care and/or nutrition guidelines can be useful, but in fact a lot of work needs to go into trainings, sensitization, and message development, for effectively putting them into practice. Psychosocial support is also needed but was/is missing in the current response, according to participants.

Messaging and sensitization to communities. A combination of mass media (e.g., radio) and interpersonal messages (e.g., community health agents going house to house; midwives giving information during childbirth) were used as channels in SBCC during the outbreak. Early on, illiteracy, distrust, misinformation, and fear hampered effective communication efforts. “*During the heart of the epidemic everything was very difficult*, *even at the mosque* …” explained one informant who articulated the magnitude of overall confusion at that time.

After more coordinated efforts that involved pilot testing as an important stage of message development, the effectiveness of SBCC improved. Interpersonal messages were more accepted due to greater trust and familiarity associated with fellow community members who passed those messages, namely community leaders, midwives, and community health agents. The health agents were particularly instrumental in passing messages from health clinics to community members, according to interviews with midwives.

Informants also explained that community-level committees were set up by the community members themselves, and proved to be a critical liaison for effective communication between the biomedical and local communities.

“*We put into place sub-committees. We chose the able-bodied youth to be trained as community agents and then they were answerable to the head of the health center…who represented the community to the health system. Decisions were coming from the head of the health center to the community health agents. Then with the supervision of the head of the health center, they (community health agents) went out every time to check the state of certain children (screening) because there were some (children) with hot skin and community agents were informed of these cases*.”

                                        Informant, Community Leader, Kindia

One participant explained that because community members, “*really trust the old traditional ways…”* referring to health and nutrition behaviors, as well as benefit from clearer explanations in local dialects, messaging through familiar, interpersonal channels was important for positive behavior changes.

Several participants gave the example of a commonly-held perception that giving a young child diverse foods will cause constipation during complementary feeding. One example of effective communications to help overcome local perceptions was sensitization through community-level trainings. These trainings reportedly were not in place early in the outbreak but did improve greatly over time and focusing on improved behaviors of WASH, appropriate acute malnutrition screening, dietary diversity during complementary feeding, feeding with RUTF, and appropriate breastfeeding practices, for example.

“*It took a major investment from [UN agency] and its partners to rebuild trust within the communities, in terms of service quality in giving higher-quality care (during the outbreak), and also in terms of demand generation so that communities would resume (typical health) consultations within the existing health structures*.”

                                        Key Informant, Management, Conakry

Some nutrition-related behavior changes persist today, according to interview data. Informants explained that handwashing practices and malnutrition screening are two primary examples of positive behaviors that have been carried over as part of typical community health practices today. On the downside, some interviews indicated that bottle feeding practices have also continued until this day.

## Discussion

Among a variety of participant types, including policy and management level professionals, as well as community and front-line workers, this qualitative study revealed salient perceptions related to the impact of EVD on health and nutrition at multiple different levels. Taken together, they have elucidated important lessons from which the humanitarian health and nutrition community can take forward for improving similar responses in the future. These findings are among the first to examine the response to Ebola in Guinea from multiple perspectives, with consideration of both qualitative and quantitative data, for generating sound recommendations (Table 4).

**Table 4:** Summary of recommendations for future nutrition responses in the context of outbreaks.

**Guidelines**
1. Emergency preparedness guidelines should outline steps for humanitarian actors, including front-line health workers, to take considering both biomedical and social spheres of medicine during a response
2. Both immediate (nutrition-specific) and underlying (nutrition-sensitive) nutritional considerations need to be core components of infectious disease outbreak response planning
3. Easy-to-use, readily available treatment and care guidelines should be disseminated among humanitarian actors in preparation for future situations warranting such guidance
**Social and Behaviour Change Communications (SBCC)**
1. Where food assistance is provided, ensure SBCC is implemented as a complementary intervention for improved acceptability and appropriate utilization
2. Developing culturally-appropriate SBCC requires formative work, which can be completed in short time periods using Focused Ethnographic Study or Rapid Assessment Procedures methodologies
3. Pre-testing communications channels and messages is a critical step prior to the implementation of SBCC activities to improve health and nutrition practices, even during emergencies
**Coordination and Collaboration**
1. Establishing trust between biomedical and local communities is a critical foundation to establish in the early stages of response efforts where health- and nutrition-seeking behaviours are important
2. The biomedical and local communities should work jointly together with shared responsibilities during response efforts through community structures and in line with the socio-cultural context
3. Where food assistance is provided, coordination and collaboration at all organizational levels should be a priority to avoid stock outs, overcome staffing shortages, and ensure consistent messaging

A primary finding from this study was that in the context of the humanitarian response to Ebola, at least in Guinea, the heavy biomedical focus, initially did not consider important social processes at work within communities and in relation to health-seeing behaviors. This finding is critical for improving responses to outbreaks in the future, with the need for a combination of approaches from both biological and social spheres of medicine [36]. Human health is complex, not only grounded in the natural sciences, but also in interconnected cultural and social tenets that drive health behaviors [37, 38]. Hewlett, an anthropologist, emphasized the complex interplay of ecology, culture, human biology and nature in Guinea to be inter-related factors that tasked humanitarians to effectively and appropriately respond to EVD in this setting [39]. Considering the local complexities, historical dimensions, social dynamics, and socio-cultural characteristics of target populations should be just as integral to response efforts as biomedical and logistics concerns. Engaging Ebola survivors during the response in Guinea was found to be one effective strategy for improving behavioral compliance at treatment centers [40]. In the context of nutrition, whose related behaviors often stem from longstanding traditions and strong cultural determinants, the importance of this consideration cannot be truer when behavior change is needed to curb such an outbreak.

This study also underscored the multi-level impact that the EVD outbreak had on nutrition specifically in Guinea. Nutrition-related impacts were felt among three important pillars of food security: availability, access, and utilization [41] as well as at basic, underlying, and immediate levels determining nutritional status [42]. While additional empirical evidence would help confirm specific nutrition-related burdens, our qualitative data shed light on some of the potential short- and long-term effects it may have had on infant and young child health, especially among orphans who lost parents to Ebola and could no longer reap breastfeeding benefits [43], or entire family units that were quarantined and unable to engage in optimal care practices that we know are critical for child nutritional health [44]. These findings relevant to nutrition may help explain the disproportionate impact of Ebola on maternal and child health [45]. Nutrition should be a core component of an infectious disease response, not considered as a standalone activity, but instead integrated into each aspect of care, treatment and recovery. The provision of specialized nutritious foods during the response in Guinea was perceived to be a critical trust building intervention bridging the local and biomedical communities, highlighting again the importance of social processes underlying even the nutrition response.

Thus, our findings serve to emphasize the important, yet delicate nature of communications and their potential consequences on health-seeking behaviors in Guinea. While communications are critical during an outbreak, they require appropriate selection of channels, pilot testing of messages, preferred media for local populations, and thorough trainings at each level of the health system. Our research indicated that there was a very low level of baseline trust among community members toward the biomedical community early on during the outbreak. Research in Liberia and Sierra Leone also found trust and fear to be key factors negatively influencing health-seeking behaviors and population health during the responses in those two countries [46–48]. Computer modeling predicted that even a 10% increase in hospital admissions during the outbreak would have reduced the length of Ebola transmission chains in Guinea by 26% (95% CI 4–45) [49]. Even the drop in OTP admissions we found in secondary data analysis is likely not a ‘reduction in caseloads’, but instead a reflection of negative changes in health seeking nutrition behaviors [50]. Effective social and behavior change communications can be critically important for improving health and nutrition in times of outbreak.

Therefore, funding should be allocated carefully, with special attention paid to ensure practitioners who understand social and behavioral dynamics are engaged early on during response efforts. Earlier engagement of trusted sources, such as traditional healers and community elders, may have led to more rapid containment of Ebola through earlier behavior change around safer, traditional burial practices [51]. Front-line workers reported a complete breakdown of social connectedness and community trust at all levels of life in Sierra Leone during the outbreak there [52]. Further, the perceived risk and fear of illness among community members may not be aligned with that of the humanitarian community so understanding a situation, perhaps through Focused Ethnographic Studies [53], while also engaging with a community, is critical. Without knowing how Ebola is cognitively represented or emotionally faced may hamper social and behavior change efforts at a time when response timeliness is so critical for population health [54].

Finally, this study highlighted the importance of having treatment and care guidelines available for organizations and front-line health staff that are easy-to-use, readily available, and acceptable for local populations. A 126-page communications toolkit, *Communication for Behavioral Impact (COMBI)*: *A toolkit for behavioral and social communication in outbreak response* [55], was made available in 2012, but none of our interview data suggested it was used in Guinea during Ebola sensitization and messaging efforts. Our findings point to the importance of wider dissemination and improved user-friendliness for situations such as outbreaks, when confusion and distrust are at their peak. The use of pictures is critical for front-line staff to use as job aides for going house to house, as an example. Further, such materials should be easily adapted to local languages with simple language structures, straightforward concepts, and material easily adapted to trainings, which we found to be a critical aspect of the sensitization efforts in this Guinea response. COMBI should be updated based on lessons learned from the experiences we have documented in this study, and incorporated as a core focus within the current guiding document entitled, *2015 WHO Strategic Response Plan*: *West Africa Ebola Outbreak* [56].

This study had several strengths. First, this multi-phased study design benefits from an iterative and emergent design, which allowed us to purposefully alter sampling procedures and interview guide content as needed during fieldwork [57]. Doing so allowed for new participant selection and question refinement that built off findings throughout fieldwork. Second, we went to great lengths purposefully sampling a variety of participant types in order to more fully address the study objective comparing multi-level perspectives [58]. Third, the study was designed to include methodological triangulation, using both primary and secondary data analysis. Triangulation is considered to be one of the most important aspects of ensuring data credibility in qualitative research [22, 59]. These three aforementioned aspects of research design are primary strengths of qualitative research and, in our study, gave us more confidence in our findings.

Limitations of this study included a sample size that did not allow for data saturation among all strata, an important consideration to guide sampling procedures in qualitative research. However, the sample sizes did allow for identification of salient themes relevant to the research questions. Also, due to the timing of the interviews after the outbreak, it was not possible to interview everyone directly involved during the crisis. As a result, many key informants interviewed had not directly used the interim guidelines and therefore could not comment directly about their utility. The emerging themes from the individuals who were interviewed, though, can be considered for improved humanitarian responses in the future and therefore are still useful.

## Conclusions

This study highlighted the multi-faceted nature of the Ebola outbreak in Guinea, which contained not only biomedical but also social and behavioral processes at various levels. While infectious disease outbreaks require urgent action for appropriate response, it is important to not underestimate the time and efforts required for gaining acceptance, buy-in, and involvement of the local community. Especially in the context of nutrition, where cases of severe malnutrition will likely be exacerbated and dietary practices are steeped in longstanding cultural traditions, behavior change can be slower than desired in the context of an emergency. This study underscores the importance of working with communities to ensure that the social and behavioral dimensions of public health are considered from the outset of a response, especially in the context of maternal and child nutrition.

## Supporting information

S1 File Key informant qualitative interview guide. (DOCX)

S2 File Informant qualitative interview guide. (DOCX)
