# Correction: Implications of the Ebola virus disease outbreak in Guinea: Qualitative findings to inform future health and nutrition-related responses

**DOI:** 10.1371/journal.pone.0204675

**Published:** 2018-10-03

**Authors:** Stephen R. Kodish, Fabian Rohner, Jean-Max Beauliere, Mamady Daffe, Mohamed Ag Ayoya, James P. Wirth, Ismael Ngnie-Teta

There is an error in the XML that is causing the fifth author’s name to be indexed incorrectly. The name should be indexed as: Ayoya MA. The correct citation is: Kodish SR, Rohner F, Beauliere J-M, Daffe M, Ayoya MA, Wirth JP, et al. (2018) Implications of the Ebola virus disease outbreak in Guinea: Qualitative findings to inform future health and nutrition-related responses. PLoS ONE 13(8): e0202468. https://doi.org/10.1371/journal.pone.0202468

In the Acknowledgments section, the final contributor’s name is spelled incorrectly. The correct name is: Meagan Harrison.
