# Supplementary material for: Implications of the Ebola virus disease outbreak in Guinea: Qualitative findings to inform future health and nutrition-related responses
Source: PLoS One. 2018 Aug 23;13(8):e0202468. doi: 10.1371/journal.pone.0202468 (PMC6107191; doi:10.1371/journal.pone.0202468)
Supplement: S1 File — (DOCX) [file pone.0202468.s001.docx]

**Interview Guide (A) – Stakeholders & Care Staff**

Understanding Key Informant Perceptions of Ebola outbreak

**Guide A Directions**: This semi-structured guide is a phase 1 guide to be used among Key Informants, including stakeholders in upper and mid-management positions. Findings from phase 1 will be used for developing a tailored version then to be used in phase 2 for Informants, include lower-level stakeholders and care staff.

**Section 1. Introduction**

- **To begin, it would be great just to first hear about your organization**
  - Probe on services, responsibilities, staff, etc.
- **Person’s description of his/her job role and responsibilities**
  - Probe on his/her specific professional expertise

Thanks, that helps me understand the organization and role better. Let’s now go into detail about work.

- **From the time you arrive at work, until when you leave, I would like you to describe your typical work day to me. Could you describe it in detail**?

That level of detail is exactly the type of response I am looking for. It is a chance for you to tell me about your experiences and perceptions in detail. I want to now transition to discussing the Ebola outbreak.

**Section 2. Ebola Impact (general impact)**

- **First, could you start by just telling me about the Ebola outbreak in Guinea**
  - Probe on personal experiences
  - Probe for personal narratives/stories
- **General impression on the impact of the EVD outbreak on any of these aspects:**
- Food security, food prices, harvest, access to markets, changes in demand
- Probe on any specific changes observed if mentioned
- Probe on where geographically these changes were observed?
- **Talk about how you feel Guinea, overall, responded to the Ebola outbreak**
  - Probe on how well/poorly the response was handled
  - Probe strengths and weaknesses of response by sector

**Section 3. Ebola Impact (on stakeholder organization)**

- **You explained the overall response to the outbreak. How about any specific impacts of the outbreak on your organization?**
  - Probe on organizational changes that occurred as a result of outbreak
  - Probe on organizational capacity available versus what was required
  - Probe on any services that had to be continued/discontinued due to EVD
  - Probe on primary sector within organization impacted by Ebola outbreak
  - Probe on funding-related impacts

**Section 4. Ebola Impact (on Health System)**

- **Now could you describe the Ebola impact on the health system, in particular**
  - Probe on any draining of or provision of additional resources
  - Probe on opening of or closing of facilities
  - Probe on impact on staffing (additional staff needed, fewer staff needed, specific types of staff required) and patient care
  - Probe on any cost impacts for health services
  - Probe on impact on drug availability

**Section 5. Ebola Impact (on nutrition services within stakeholder organization)**

- **Let’s now focus on nutrition specifically. Could you explain the specific impact of the Ebola outbreak on nutritional health services provided by your organization?**
  - Probe on any changes that occurred
  - Probe on capacity and technical support available versus what was required
  - Probe on any services continued/discontinued due to Ebola
  - Probe on funding-related impacts related to nutrition specifically
- **What was the number one primary challenge to address nutritional health and underlying reasons?**
  - Probe on coping strategies used by the organization to overcome primary challenge
  - Probe on stakeholder confidence in staff to address the nutrition-related impacts
- **Perception of how typical his/her organization’s experience compared to those of others**
  - Probe on reasons why/why not with examples

**Section 6. General Support for Nutritional Health**

- **Discuss for me your perception of the quality of support given to nutritional health services in Guinea during the outbreak.**
  - Probe on whether response was adequate/inadequate and reasons why
  - Probe on what specifically he/she wished had been more supported and how
  - Probe on quality of response at centralized and decentralized levels
  - Probe on level of recognition that nutrition was a critical aspect for effective Ebola response

**Section 7. Nutritional Support using Interim Guidelines**

- **Overall, could you explain the quality of nutritional care provided using the interim guidelines provided by the global community?**
  - Probe for reasons why quality good/not good with examples
  - Probe on level of acceptability of the care provided to patients
  - Probe on level of community trust toward the biomedical community
- **The nutrition sector provided care at treatment centers and clinics and should have been guided by the guidelines *“Nutritional Care of Children and Adults with Ebola Virus Disease in Treatment Centers”*. Could you talk about your organization’s utilization of these guidelines for patients?**
  - - Probe on how they were used by staff during implementation (if at all)
    - Probe on facilitating factors and barriers to implementation
    - Probe on usage and level of acceptance by care staff
    - Probe on any other strategies used or guidelines followed by care staff instead
  - Probe on key lessons learnt from the implementation of these guidelines
- **Fortunately, some patients recovered. In those cases, the nutrition sector provided support for Ebola survivors and may have been guided by Joint Guidance for Nutritional support*.* Could you talk about your organization’s utilization of these guidelines for survivors?**
  - - Probe on how they were used by staff during implementation (if at all)
    - Probe on facilitating factors and barriers to implemention
    - Probe on level of usefulness for Ebola survivors
    - Probe on any other strategies used or guidelines followed by partner organizations
  - Probe on key lessons learnt from the implementation of these guidelines

- **Also, guidelines around Infant & Young Child Feeding practices were provided for the nutrition sector to provide support for caregivers. Could you talk about your organization’s utilization of these guidelines for feeding in the Ebola context?**
  - - Probe on how they were used by staff during implementation (if at all)
    - Probe on facilitating factors and barriers to implementing them
    - Probe on adequacy of guidelines for addressing all feeding-related challenges and inquiries in the Ebola context among health workers, welfare services, and community members
    - Probe on any other strategies used by community instead of these guidelines
    - Probe on level of acceptability of these guidelines by community members
  - Probe on key lessons learnt from the implementation of these feeding guidelines

**Section 8. Coordination and Information Sharing**

- **Could you talk about the ease of coordination during the Ebola outbreak?**
  - Probe on coordination among partners to help with implementation of nutritional guidelines outlined above
- **Please explain availability of necessary information for organization’s ability to address outbreak.**
  - Probe on stakeholder confidence in finding information or capacity support when needed?
  - Probe on what was needed organizationally to help develop better M&E during outbreak
  - Probe on overall quality and timeliness of resources and support sent from global levels

**Section 9. Recommendations & Lessons Learned**

- **What are your key lessons learned from this specific outbreak?**
  - Probe on nutrition response lessons learned
- **Could you talk about how you would like to see global or country-level resources and support better respond to such an outbreak?**
- **What would be a recommended strategy to improve coordination among stakeholders in an event such as this one?**
- **Considering everything, what are your top recommendations for improving the overall response to such an outbreak like this in the future?**
  - Probe on recommendations to improve health system
  - Probe on ways to better engage with health and other sectors to ensure nutrition gets the same priority and support as other areas

**Thank you so much. That information was really helpful. Do you have any questions for me?**

**Demographics:**

1. **Gender**
2. **Type of organization**
3. **Specific job/role**
4. **Years in his/her role**
5. **Geographic region in Guinea**
