# Supplementary material for: Implications of the Ebola virus disease outbreak in Guinea: Qualitative findings to inform future health and nutrition-related responses
Source: PLoS One. 2018 Aug 23;13(8):e0202468. doi: 10.1371/journal.pone.0202468 (PMC6107191; doi:10.1371/journal.pone.0202468)
Supplement: S2 File — (DOCX) [file pone.0202468.s002.docx]

**Interview Guide (B) – Frontline Care Staff & Beneficiaries**

**Understanding Informant Perceptions of Ebola outbreak**

**Guide B Directions**: This semi-structured guide is a phase 2 guide to be used among Informants, including frontline care staff and beneficiaries.

**Section 1. Introduction**

- **To begin, it would be great just to first hear about your community**
  - Probe on typical livelihoods, challenges, family
- **Person’s description of his/her family members**
  - Probe on his/her children, children’s ages, etc.

- **From the time you get up in the morning, until when you go to bed, I would like you to describe your typical day to me. Could you describe it in detail**?

That level of detail is exactly the type of response I am looking for. It is a chance for you to tell me about your experiences and perceptions in detail. I want to now discuss the Ebola outbreak.

**Section 2. Ebola Impact (general impact)**

- **First, could you start by just telling me about the Ebola outbreak in this area**
  - Probe on the extent/gravity of EVD in this area
  - Probe on personal feelings toward the outbreak
  - Probe for personal narratives/stories
  - Probe on person’s understanding of the Ebola Virus – including:
    - signs/symptoms
    - causes
    - modes of transmission
    - prevention/treatments
    - prognosis
- **What were some of the biggest challenges your community dealt with during the outbreak?**
  - Probe on strategies the community used to overcome the challenges and their effectiveness
- **Could you explain generally how well you feel the federal and global health communities responded to the outbreak here?**
  - Probe on specifics related to how well/how poorly the response was
  - Probe on the perceived level of response by sector

**Section 3. Perceptions of Nutrition Care to Ebola Patients**

- **The nutrition sector provided care at treatment centers and clinics. Could you tell me about what types of nutrition care and support were provided to Ebola patients?**
  - Probe on differences of care by type of patient (illness severity, level of condition gender, age)
- **Could you explain the quality of that nutritional care?**
  - Probe for reasons why quality good/not good with examples
  - Probe on perceived effectiveness of the quality of nutrition care
  - Probe on level of acceptability of the care provided to patients
  - Probe on level of community trust toward the biomedical community
- **Were there some organizations that were providing better nutritional care to patients?**
  - Probe for specific organizations and reasons why some better than others

**Section 4. Perceptions of Nutrition Support to Ebola Survivors**

- **Fortunately, some people recovered from Ebola. Could you talk about the primary reasons some people got better and others did not?**

- **Could you explain to me the level of nutritional support Ebola survivors you know were given after they recovered from Ebola?**
  - Probe on what specific nutrition support was provided
  - Probe on perceived effectiveness/usefulness of this nutrition support
  - Probe for ways to make this support for survivors more useful and helpful after recovery
- **Were there some organizations that were providing better nutritional support to survivors?**
  - Probe for specific organizations and reasons why some better than others

**Section 5. Ebola Impact on Infant & Young Child Feeding**

- **First, could you start by just telling me about how Ebola impacted infant and young child feeding practices**
  - Probe for specific changes illustrated by personal narratives/stories
  - Probe for specific challenges and coping strategies
- **Maybe you are familiar with the Ready to Use Infant Formula provided to some families for feeding infants in the context of Ebola outbreak. How did you feel about this product’s effectiveness?**
  - Probe on its need and appropriateness during the outbreak in this context
  - Probe on effectiveness in comparison to breast milk
  - Probe on perceived safety of using it
  - Probe on overall acceptability of caregivers and community
- **Explain how you/your close ones received useful information/messages regarding infant and young child feeding during the Ebola outbreak.**
  - Probe on ease/difficulty of getting this information
  - Probe on cultural appropriateness of this information (considering literacy, pictures, media channels, etc.)
  - Probe on level of acceptance of the information/messages
  - Probe on the messages/information helped overcome specific feeding challenges
- **Could you tell me to what extent these messages and information were effective in changing your infant and young child feeding practices during Ebola outbreak?**
  - Probe on any multi-level barriers and facilitating factors to behavior change
    - **Intrapersonal** (knowledge, attitudes)
    - **Interpersonal** (influences of others, in-laws, neighbors)
    - **Household dynamics** (other children of similar age, head of household not permitting new practices)
    - **Community** (SES, cultural norms)
    - **Structural** (distance to health clinic)
- **Now that the Ebola outbreak is over, could you talk about how infant and young child feeding practices are today?**
  - Probe for any lasting feeding changes based on messaging given during outbreak
  - Probe on reasons for reverting back to old, traditional practices (if this happened)
  - Probe on key feeding messages taken from the Ebola outbreak and still used today among caregivers and households
    - Probe for reasons some messages worked for sustaining behavior change and others did not work as well

**Section 6. Recommendations & Lessons Learned**

- **You talked a lot about your experiences - thank you. On a final note, I would love to hear your recommendations for ensuring that such a response is more effective and appropriate for your community in the future, or another community?**
  - Probe for specific actions to be put in place and why
  - Probe whether any are community-specific or could be generalizable to other communities
- **Could you describe specific recommendations for strengthening the nutrition support and services during an outbreak such as this one, specifically?**
  - Probe on any suggestions for improving nutritional care to patients
  - Probe on any suggestions for improving nutritional support to survivors
  - Probe on any suggestions for helping caregivers safely feed infants during such an outbreak
  - Probe on any suggestions for improving communications/messaging related to during an outbreak such as this one.

**Thank you very much for your time and information. Do you have any final comments or questions?**

**Demographics:**

1. **Gender**
2. **Type of organization**
3. **Specific job/role**
4. **Years in his/her role**
5. **Geographic region in Guinea**
